# HOPX Plays a Critical Role in Antiretroviral Drugs Induced Epigenetic Modification and Cardiac Hypertrophy

**DOI:** 10.3390/cells10123458

**Published:** 2021-12-08

**Authors:** Shiridhar Kashyap, Maryam Rabbani, Isabela de Lima, Olena Kondrachuk, Raj Patel, Mahnoush Sophia Shafiei, Avni Mukker, Aishwarya Rajakumar, Manish Kumar Gupta

**Affiliations:** Division of Metabolic and Cardiovascular Sciences, Burnett School of Biomedical Sciences, College of Medicine, University of Central Florida, Orlando, FL 32827, USA; fn288641@ucf.edu (S.K.); Maryam.rabbani@ucf.edu (M.R.); isabelafuturo@knights.ucf.edu (I.d.L.); Olena.Kondrachuk@Knights.ucf.edu (O.K.); rpatel4001@Knights.ucf.edu (R.P.); sophiash@berkeley.edu (M.S.S.); avnimukker@Knights.ucf.edu (A.M.); aishu.rajakumar@Knights.ucf.edu (A.R.)

**Keywords:** cardiomyocytes, antiretroviral therapy, hypertrophy, HOPX, epigenetics, HDAC inhibitor

## Abstract

People living with HIV (PLWH) have to take an antiretroviral therapy (ART) for life and show noncommunicable illnesses such as chronic inflammation, immune activation, and multiorgan dysregulation. Recent studies suggest that long-term use of ART induces comorbid conditions and is one of the leading causes of heart failure in PLWH. However, the molecular mechanism of antiretroviral drugs (ARVs) induced heart failure is unclear. To determine the mechanism of ARVs induced cardiac dysfunction, we performed global transcriptomic profiling of ARVs treated neonatal rat ventricular cardiomyocytes in culture. Differentially expressed genes were identified by RNA-sequencing. Our data show that ARVs treatment causes upregulation of several biological functions associated with cardiotoxicity, hypertrophy, and heart failure. Global gene expression data were validated in cardiac tissue isolated from HIV patients having a history of ART. Interestingly, we found that homeodomain-only protein homeobox (HOPX) expression was significantly increased in cardiomyocytes treated with ARVs and in the heart tissue of HIV patients. Furthermore, we found that HOPX plays a crucial role in ARVs mediated cellular hypertrophy. Mechanistically, we found that HOPX plays a critical role in epigenetic regulation, through deacetylation of histone, while the HDAC inhibitor, Trichostatin A, can restore the acetylation level of histone 3 in the presence of ARVs.

## 1. Introduction

According to a recent study published by the World Health Organization, 37.7 million people are living with HIV infection globally, and almost 27.7 million people have access to antiretroviral drugs (ARVs) [1]. Due to the efficacy of antiretroviral therapy (ART), viral replication is inhibited, and the viral load becomes undetectable in the patients’ serum. However, people living with HIV (PLWH) have to commit to ARVs for the rest of their life to suppress viral reactivation [2,3]. Successful application of ART has significantly improved life expectancy in the PLWH and turned HIV from a terminal disease into a chronic disease. PLWH show an onset of non-HIV-related symptoms such as chronic inflammation, an unusual immune system, and a higher risk of cardiovascular disease (CVD) [4,5,6]. The relative risk of various CVDs in HIV patients has tripled since HIV was first reported, with a 61% increase in relative risk of CVDs, such as ischemic stroke, heart failure (HF), pulmonary hypertension, myocardial infarction, and venous thrombosis [7].

Initially, PLWH were treated with various regimens of ARVs, starting with a nucleoside reverse transcriptase inhibitor (NRTI) azidothymidine (AZT); however, in 1995, the FDA recommended the use of a combination of protease inhibitors along with NRTI, as a cocktail [8]. In the past three decades, various treatment regimens have been introduced and replaced with more effective antiretroviral drugs, including integrase inhibitors, CCR5 blockers, and antibody therapy [9]. According to a recent recommendation of the International Antiviral Society-USA panel, a cocktail of three drugs, including two nucleoside reverse transcriptase inhibitors and an integrase inhibitor, can be used to suppress viral replication [10]. However, some underdeveloped countries still use the old regime of drugs, including NRTIs and protease inhibitors, as a first-line drug for the treatment of HIV [11,12,13]. Although, ART improves the life expectancy of PLWH, the development of CVD in HIV patients is responsible for a higher mortality rate. It is estimated that 73% of HIV-infected patients will show CVD at the age of 50 by the year 2030 [14]. Recent studies suggest that ART therapy causes the induction of weight gain and fat deposition in HIV patients, leading to dysregulation of metabolism and development of metabolic disease and cardiac hypertrophy [15,16]. Additionally, it was found that 6.5% of HIV patients accessing ART therapy have abnormal cardiac structure, along with left ventricular hypertrophy [17,18]. An ongoing study with HIV, characterizing heart function on antiretroviral therapy (CHART), suggests that HIV patients under ART therapy can develop cardiac dysfunction, due to multiple complications, such as mitochondrial toxicity, endoplasmic reticulum stress, inflammation, and metabolic disease [19]. However, the underlying molecular mechanism that triggers cardiac remodeling, leading to heart failure, mostly remains unclear.

Our recent study found that ARV treatment to cardiomyocytes causes viability loss and induction of cellular hypertrophy (31). To elucidate the mechanism of ARV-mediated cardiomyocytes hypertrophy, we treated the primary cardiomyocytes with the ARVs, and global transcriptome was analyzed by RNA-sequencing (RNA-seq). Our data show that ARVs induced several pathological gene expressions, including, apoptosis, cardiomyocytes hypertrophy, fibrosis, and heart failure. Interestingly, we found that expression of a cardiac co-transcription factor, Homeodomain only-protein homeobox (HOPX), was significantly upregulated in cardiomyocytes due to ARV treatment. HOPX is an important non-DNA binding co-transcription factor that regulates cellular proliferation and development of the heart [20,21,22]. Furthermore, it was found that HOPX regulates cellular transcription through interactions with other cellular transcription factors such as serum response factor (SRF) and histone deacetylase protein (HDAC), and modulates the various stress signaling pathways, leading to cardiac hypertrophy and heart failure [23,24].

Our study found that ARVs induce differential gene expression associated with biological processes and pathways, leading to cardiac hypertrophy. Furthermore, through a loss-of-function and gain-in functional approach, we found that HOPX plays a critical role in the ARV-mediated epigenetic modification and induction of cardiomyocyte hypertrophy.

## 2. Materials and Methods

### 2.1. Cells Culture and ARVs Treatment

Animal studies were approved by the Institutional Animal Care and Use Committee of the University of Central Florida. In this study, we used neonatal rat ventricular cardiomyocytes (NRVCs). Cells were isolated from 2–3-day-old Harlan Sprague–Dawley rats (Jackson Laboratory, Bar Harbor, ME, USA), as described previously [25]. In brief, left ventricles of 2–3-day-old rat pups were isolated and digested overnight with 0.05% trypsin at 4 °C, followed by collagen treatment for 40 min at 37 °C. Cardiomyocytes were separated by pre-plating the digested cell suspension. Initially, NRVCs were grown in Minimum Essential Media α (Gibco, Grand Island, NY, USA), having 10% fetal bovine serum (FBS) and 1X anti-anti (Gibco) for 24 h. For protein isolation, cells were grown in 10 cm plates at a density of 1.5 × 10^6^ cells/plate, and for RNA isolation cells were grown in 6-well plates at a density 1 × 10^5^ cells per well. Experiments were performed with NRVCs grown in Dulbecco’s modified Eagle’s essential medium (DMEM) (Gibco) supplemented with 2% FBS, 1X anti-anti for at least 24 h. To test the effect of ARVs, NRVCs were treated with 5 µM ARVs (Ritonavir, Abacavir, Atazanavir, and Lamivudine, (Selleck Chemicals Llc., Pittsburgh, PA, USA)) for different times: 4, 12, and 24 h, according to the experimental set up. The treatment concentration of ARVs was selected according to the drug levels in the patient’s plasma [26]. For adenovirus-mediated overexpression, cells were incubated with adenovirus (1:1 pfu) in serum-free DMEM for 2 h, and then the cells were grown in DMEM with 2% FBS for the next 48 h. ARVs treatment was started after 48 h of adenovirus infection. Control cells were infected with adenoviruses encoding a green fluorescent protein (Ad-GFP) or adenovirus null (Ad-null) (Vector Biolabs, Malvern, PA, USA). For the preparation of HOPX adenovirus, mouse cDNA for Hopx (Origene, Rockville, MD, USA) gene was cloned in the pShuttle vector, and adenovirus was generated using AdEasy adenoviral vector systems (Agilent, Santa Clara, CA, USA).

### 2.2. Global Transcriptomic Profiling

For RNA isolation, cardiomyocytes were treated with ARVs and control cells were treated with DMSO in three replicate groups; total RNA was isolated using an RNeasy Mini kit (Qiagen, Germantown, MD, USA), according to the manufacturer’s instructions. RNA was quantified by Nanodrop 8000 (Thermo Fisher Scientific, Waltham, MA, USA), and RNA quality and integrity were assessed with Agilent Bioanalyzer (Agilent), and 2 μg RNA with integrity Number (RIN) >7 was subjected to RNA sequencing (Novogene, Sacramento, CA, USA). Global transcriptomic profiling was performed following a standard protocol. In brief, first and second strand synthesis was performed using a TruSeq RNA Sample Preparation Kit (Illumina, San Diego, CA, USA). cDNA fragments ~400 bp were obtained with gel electrophoresis, followed by PCR amplification. cDNA library was sequenced using Illumina’s HiSeq 2000 with four RNA-seq libraries per lane. Filtered and sequenced reads were mapped using HISAT2 algorithms, with the reference genome Rattus norvegicus release 97. The quantity of total mapped reads and the percentage of clean reads were calculated, including the quantity of multiple mapped reads and the percentage of clean reads, and the quantity of uniquely mapped reads and the percentage of clean reads. The total mapped reads of fragments larger than 70%, and multiple mapped reads or fragments no more than 10% were accepted. Transcripts were assembled using StringTie v1.3.3b. CuffCompare v2.1.1 was used to compare transcripts with default settings. In RNA-seq experiments, the gene expression level was estimated by the read count, mapped to the genome or exon. Read counts are proportional to gene expression level, gene length, and sequencing depth. Fragments per kilobase of transcript sequence per million base pairs sequenced (FPKM) is the most common method of estimating gene expression levels, which takes into consideration the effects of both sequencing depth and gene length when counting of fragments [27,28]. Differentially regulated genes for NRVC treated with ARV were identified using the DESeq2 R package [29]. Statistical routines for determining differential expression in digital gene expression data were performed using a model based on the negative binomial distribution, and the resulting *p* values were adjusted using Benjamini and Hochberg’s approach for controlling false discovery rate. Read count was adjusted using trimmed mean of M-values (TMM), then a differential expression analysis was performed using the EdgeR package. The threshold of differential expressed genes was considered padj <0.05. The raw RNA-seq data were deposited in the Gene Expression Omnibus database (accession number: GSE188210).

### 2.3. Functional Annotation

Gene ontology (GO) terms for molecular processes, cellular components, and biological processes enrichment analysis of differentially expressed genes were implemented using the clusterProfiler R package. GO terms with padj <0.05 were considered significantly enriched with differentially expressed genes [30]. Differentially expressed genes with ARVs were subjected to the ClusterProfiler R package, to test the statistical enrichment in KEGG pathways. Disease and bio-function, as well as association with cardiotoxicity, were accessed using ingenuity pathway analysis (IPA) (Qiagen).

### 2.4. RNA Isolation and Expression Analysis by Quantitative Real-Time PCR 

RNA from cardiomyocytes was isolated using an RNeasy Mini kit (Qiagen), as described in the kit manual. RNA from the cardiac tissue was isolated using TRI reagent (Sigma, St. Louis, MO, USA). RNA samples were treated with RNase-free DNase (Qiagen) to remove DNA contamination. cDNAs were synthesized with 500 ng RNA using a SuperScript III First-Strand Synthesis SuperMix reagent kit (Thermo Fisher Scientific, Waltham, Hercules, California MA, USA). Gene expression was analyzed by qRT-PCR (QuantStudio 7 Flex), using SYBR Green master mix (Thermo Fisher Scientific) with gene-specific primers. Data were normalized with GAPDH or Actin as an internal control. 

### 2.5. Protein Extraction and Western Blot Analysis

Cells were lysed with RIPA buffer (50 mM Tris-HCl pH-8.0, 150 mM NaCl, 1% IGEPAL, 12 mM sodium deoxycholate, 1% SDS) and 1× mammalian protease inhibitor (Sigma). Next, cells were sonicated for 20 s, using a 2 s on and 2 s off setting, at 25% amplitude power (Qsonica, Newtown, CT, USA). Protein extract was centrifuged at 10,000 *g* for 10 min at 4 °C, and the supernatant was collected and stored at −80 °C for future use. Protein concentration was measured using a Bicinchoninic acid assay (BCA) kit (Thermo Fisher Scientific). Protein expression was determined by Western blot. Protein samples were prepared in 1× Laemmli buffer and resolved in sodium dodecyl-sulfate polyacrylamide gel electrophoresis (SDS-PAGE) (Bio-Rad, Hercules, CA, USA). Resolved proteins were then transferred to PVDF membrane with the Trans-Blot^®^ Turbo™ transfer system (Bio-Rad). Membranes were blocked with Li-Cor blocking buffer (Intercept™ Blocking Buffer, Li-COR, Lincoln, NE, USA) for 1 h at room temperature (RT) and incubated overnight at 4 °C with primary antibody. The membranes were then probed with secondary antibody (IRDye^®^ 680, red and 800, green, Li-COR) at RT for 2 h after washing with PBST twice and PBS once. Li-Cor scanner (Li-COR) was used to scan the membrane with software Image Studio ver. 4.0 (Li-COR). The antibodies used for immunoblotting were total acetylated lysine, histone 3 (Cell signaling, Danvers, MA, USA), HOPX (Thermo Fisher Scientific), H3K9ac, H3K27ac (Abcam, Waltham, MA, USA), GAPDH, β-Actin, and HOPX (Proteintech, Rosemont, IL, USA).

### 2.6. siRNA-Mediated Knockdown of HOPX 

To knockdown HOPX expression, NRVCs were treated with siRNA (Rn01_00076610, Sigma-Aldrich, St. Louis, MO, USA) using Lipofectamine 2000 (Thermo Fisher Scientific) in optimum media (Thermo Fisher Scientific) for 3 h, and then cells were grown in 2% DMEM for 24 h. After 24 h of siRNA incubation, NRVCs were treated with ARVs or Trichostatin-A (TSA) (Cell signaling) for the next 24 h. Similarly, control cells were incubated with the negative siRNA (Thermo Fisher Scientific) and treated with ARVs and TSA. 

### 2.7. Immunofluorescence Staining

Immunocytochemistry was performed in NRVCs plated on chamber slides. Cells were fixed with 4% paraformaldehyde (PFA) for 10 min at RT. Cells were washed twice with PBS and permeabilized with 0.5% Triton X-100 for 10 min at RT. After washing twice with PBS, cells were masked with 0.1 M glycine for 30 min at RT. Blocking was performed for 1 h using blocking buffer (1% BSA, 0.1% Tween 20 in PBS). Slides were then treated with HOPX antibody (Thermo Fisher Scientific) at 1:200 dilution in blocking buffer and incubated overnight at 4 °C. After washing with PBS, cells were probed with a secondary antibody labeled with Alexa Fluor™ Goat anti-mouse 488 (Invitrogen, Waltham, MA, USA) in blocking buffer at 1:200 dilution for 2 h at RT. Cells were again blocked in blocking buffer for 1 h and incubated with the second primary antibody, HDAC2 (Active Motif, Carlsbad, CA, USA) at 1:1000 dilution for 2 h. Cells were washed with PBS and probed with Alexa Fluor™ Goat anti-mouse 594 (Invitrogen) at 1:100 dilution for 2 h. Cells were mounted with VECTASHIELD HardSet antifade mounting medium with DAPI (Vector Laboratories, Burlingame, CA, USA). Images were captured using a Keyence microscope (BZ-X800, Keyence, Osaka, Japan).

### 2.8. Immunohistochemistry

Immunohistochemistry (IHC) was performed using an ABC immunoperoxidase staining kit (Vector Laboratories, Burlingame, CA, USA). In brief, cardiac tissue sections were deparaffinized using xylene. Antigen retrieval was performed in a microwave for 20 min with 1X citrate buffer (Vector Laboratories). Endogenous peroxidase activity was prevented by treating the tissue sections with 3% hydrogen peroxide. Tissue sections were blocked with the 2.5% normal horse serum (Vector Laboratories) at RT for 1 h. Tissue sections were incubated with the HOPX primary antibody (Thermo Fisher Scientific) at 1:500 dilution for 1 h at RT. Slides were washed with the 1X TBST and incubated with biotinylated anti-rabbit antibody for 30 min and then developed with 3, 3, 0-diaminobenzidine (Dako, Santa Barbara, CA, USA). Furthermore, tissue sections were counterstained with hematoxylin. Images of the stained slides were captured using a BZ-X800 Keyence microscope (Keyence).

### 2.9. HDAC Activity Assay

HDAC activity was measured with an HDAC assay kit (Abcam), according to the manufacturer’s instructions. Nuclear protein was isolated from cardiomyocytes treated with the ARVs (5 µM) and TSA (50 nM) for 12 h, as per instructions provided in the assay kit manual. HDAC activity assay was performed by mixing assay buffer, fluoro-substrate peptide, and developer. The reaction was initiated by adding 30 μg protein lysate and incubating for 30 min in black transparent bottom 96-well-plates (Thermo Fisher Scientific). For comparison, the negative control reaction was set without enzyme or with HDAC inhibitor TSA. The positive control reaction was set with crude HDAC provided in the kit. The fluorescent intensity was measured using a spectrophotometer (Bio-Tak, Synergy 4, Winooski, VT, USA) at 350 nm excitation and 450 nm emission.

### 2.10. Data Analysis and Statistical Procedures

All the experiments were performed three times or more. Statistical analyses were performed using Prism GraphPad 8.0. The results are presented as mean ± standard deviation. An unpaired Student t-test was performed for statistical significance between the control and test groups. A *p*-value < 0.05 was considered to be significant. 

## 3. Results

### 3.1. ARVs Treatment Cause Pathological Gene Expression in Cardiomyocytes

**Figure 1:**
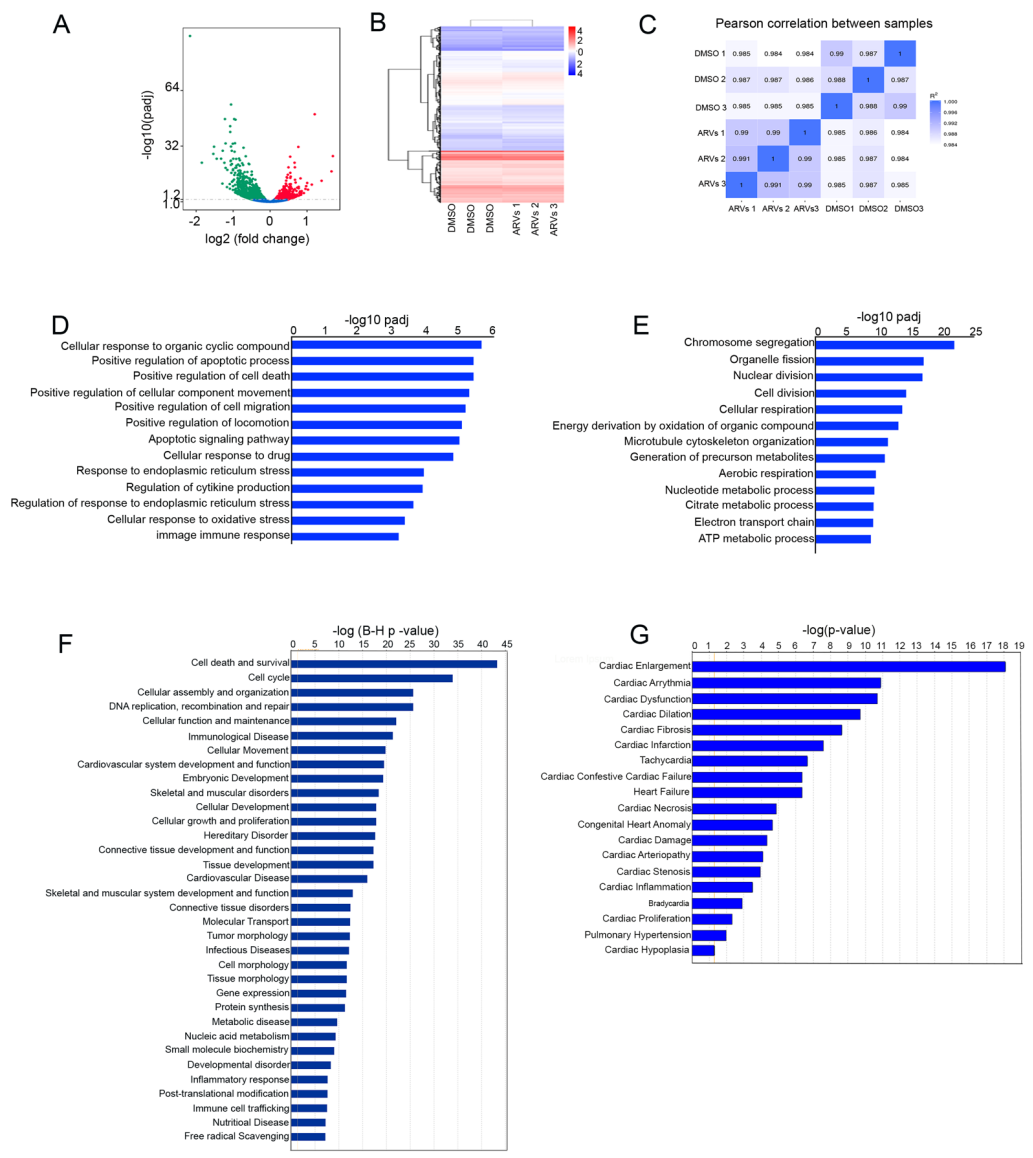
RNA sequencing analysis reveals that ARV treatment causes differential gene expression in cardiomyocytes. NRVCs were treated with a combination of ARVs (Ritonavir, Abacavir, Atazanavir Lamivudine (5 µM), and control cells were treated with DMSO for 24 h in groups of three replicates (n = 3), and total RNA sequencing was performed on the Illumina platform. ARVs treatment differentially regulates the expression of 1756 genes in cardiomyocytes. (**A**) Volcano plot shows differentially expressed genes (padj <0.05) using log2 (fold change) against-log10 (padi). (**B**) Hierarchical clustering and heatmap of differentially expressed genes show the expression level of genes in each replicate. Red color indicates genes with high expression levels, whereas blue color indicates genes with low expression levels. (**C**) Pearson correlation analysis between replicates shows a correlation coefficient between and among the replicates. The color from light to dark blue represents the increasing value of R^2^. (**D**) Differentially expressed genes were subjected to functional enrichment analysis on ClusterProfiler v2.4.3. The graph shows the relation of differentially expressed genes with biological processes. Gene ontology (GO) terms with padj < 0.05 were accepted as significant enrichment. (**E**) The graph shows downregulated biological processes during ARV treatment in cardiomyocytes. (**F**) The graph shows that the differentially expressed genes at IPA and the significance of enrichment were padj <0.05. Differentially expressed genes enriched with various disease and bio-function terms, including the cardiovascular system, development, and cardiovascular disease. (**G**) Cardiotoxicity analysis by IPA showed that the cardiac enlargement term was the top toxicity group among the differentially expressed genes in cardiomyocytes.

In our previous study, we found that ARV treatment increased cardiomyocytes’ cell size and reduced cellular viability [31]. To understand the molecular mechanism of ARV-induced cellular toxicity and cardiac hypertrophy, we performed global transcriptomic expression profiling, using RNA sequencing in NRVCs treated with a cocktail of ARVs (Ritonavir, Atazanavir, Abacavir, and Lamivudine) at a concentration of 5 µM for 24 h and compared with DMSO-treated control cells. RNA sequencing analysis showed that ARV treatment differentially regulates the 1756 genes (padj < 0.05) expressed in cardiomyocytes. A total of 701 genes were upregulated in drug-treated cells. In contrast, 1055 gene expressions were downregulated in cardiomyocytes treated with ARVs (Supplementary Table S1). Using RNAseq data, we prepared a volcano plot (Figure 1A) to show the differentially expressed genes and a hierarchical clustering heatmap to show the transcriptomic similarity between the replicates (Figure 1B). Additionally, we generated a Pearson correlation analysis among the samples, to show the association between replicates of the study groups (Figure 1C). To identify biological processes affected by the ARV treatment, differentially regulated genes were analyzed using clusterProfiler v.2.3 [30]. Biological processes with a value of padj >0.05 were considered to have a significant association with ARV treatment in cardiomyocytes. Our analysis showed that significantly upregulated genes were linked with the biological processes associated with apoptosis, cellular movement or locomotion, cellular stress, and immune response. In contrast, downregulated genes were involved in cell division and metabolism (Figure 1D,E) (Supplementary Tables S2 and S3). These results indicate that ARV treatment modulates cellular function, through the regulation of genes associated with energy metabolism, cellular division, and cell death. Earlier studies showed that various regimens of ARV treatment in HIV patients may accelerate cardiovascular disease, such as heart failure [32,33]. Our previous study also showed that ARV treatment induces hypertrophy in cardiomyocytes [31]. Furthermore, to investigate the association of differentially expressed genes with bio-function, disease, and cardiotoxicity, differentially expressed genes were analyzed using IPA software (Qiagen). IPA analysis of the differentially expressed genes showed an association with cardiovascular disease, as the top-listed disease and bio-function terms (Figure 1F). Interestingly, our data show that cardiac enlargement was the most significantly enriched cardiotoxicity term associated with differentially expressed genes (Figure 1G). Considering our investigation of ARV treatment promoting cardiomyocyte cell size [31] by cardiotoxicity analysis, we aimed, in this study, to investigate the role of genes related to cardiac hypertrophy in our data set. The IPA-mediated cardiotoxicity analysis showed that ARV treatment in cardiomyocytes causes differential expression of 135 and 73 genes associated with cardiac enlargement and hypertrophic processes (Supplementary Table S4). 

### 3.2. HOPX Is Upregulated in Cardiomyocytes and in Cardiac Tissue of HIV Patients after ARV Treatment

**Figure 2:**
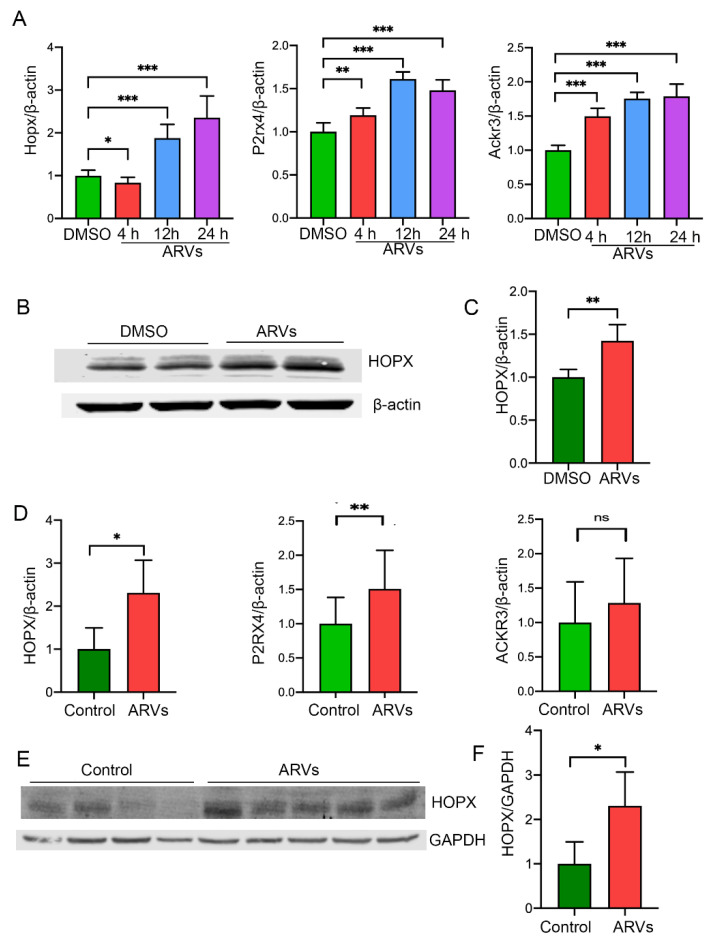
ARV treatment promotes HOPX expression in cardiomyocytes and human cardiac tissue. NRVCs were treated with 5 uM ARVs (combination of Ritonavir, Atazanavir, Abacavir, and Lamivudine) for 4, 12, and 24 h and the expression of transgenes was estimated by qRT-PCR. The expression of drug-treated cells was compared with DMSO-treated control cells. Fold change of expression was calculated against internal control β-Actin. Values are presented as means ±SDs from three biological replicates and two technical replicates each time. (**A**) Graphs show that expression of Hopx, Ackr3, and P2rx4 genes was significantly upregulated in ARVs treated cells. (**B**,**C**) Cells were treated with ARVs for 24 h, and Western blot was performed with HOPX antibody. The graph shows a quantification of the Western blot image. β-Actin was used as a loading control. (**D**) Graphs show mRNA expression of *ACKR3*, *HOPX*, and *P2RX4* genes in ART-treated heart tissue collected from HIV-positive patients (n = 8). For comparison, we included healthy controls (n = 8). (**E**,**F**) To check the expression of HOPX protein in ART-treated cardiac tissue of HIV patients, Western blot was performed. Western blot images show the increased protein level of HOPX in ART-treated tissue. The graph shows a quantification analysis of Western blot against internal control GAPDH. (*p*-value, ns = not significant; *, <0.05; **, <0.01; ***, <0.001).

Our data show that ARV treatment upregulates the expression of genes involved in cellular stress signals in cardiomyocytes (Figure 1D,G), and cellular stress is reported to be associated with cardiac hypertrophy [34]. Furthermore, to validate the expression of hypertrophy-related gene expression in ARVs treated cardiomyocytes, we selected Hopx, Ackr3, and P2rx4 genes, based on their significance and the relatedness to their role in cardiac hypertrophy. RNA sequencing results were validated using qRT-PCR, using gene-specific primers (Supplementary Table S5). Our data show that the expression of Hopx, Ackr3, and P2rx4 genes was significantly upregulated in drugs-treated cardiomyocytes (Figure 2A). Furthermore, we selected the Hopx gene to determine its role in ARVs induced cellular hypertrophy. We treated the NRVCs with the ARVs for 24 h, and the expression of HOPX protein was determined by Western blotting. Consistent with the qRT-PCR data, the expression of HOPX protein was significantly upregulated in ARV-treated cells (Figure 2B,C). Furthermore, RNA sequencing data were validated with heart tissues collected from ARV-treated HIV patients (Supplementary Table S6). Consistent with the in vitro data, our qRT-PCR data show that HIV patients have a significantly increased expression of *HOPX*, *ACKR3*, and *P2RX4* genes (Figure 2D). We also analyzed the expression of HOPX protein in the cardiac tissue by Western blotting. Our Western blot analysis showed that the HOPX expression was significantly increased in HIV patients compared to healthy controls (Figure 2E,F). 

### 3.3. HOPX Differentially Localized in the ART-Treated Cardiac Tissue

**Figure 3:**
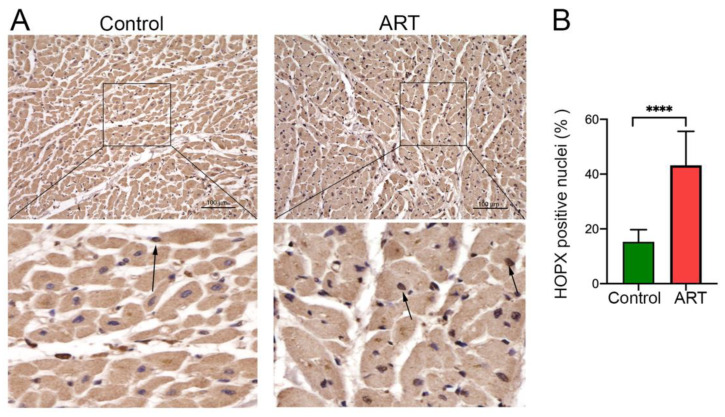
ARVs induce the accumulation of HOPX protein in the nucleus of cardiomyocytes. (**A**) Immunohistochemistry was performed in human heart tissue of HIV patients (n = 7) and healthy controls (n = 8) using HOPX antibody. The brown color represents the HOPX staining. (**B**) Graph showing percentage of brown HOPX nucleus distribution in cardiomyocytes compared to healthy controls (*p*-value ****, <0.0001).

We performed immunohistochemistry to detect the localization of HOPX protein in the cardiomyocytes of HIV patients on ART. Representative images show the localization of HOPX protein in the cytoplasm, as well as in the nucleus of the cardiac tissue. Interestingly, microscopy images also show that the distribution of HOPX protein in cardiac tissue of HIV patients was more concentrated in the nucleus compared to the healthy controls (Figure 3A,B). Taken together, our data show that the ARVs modulate the HOPX expression in cardiomyocytes, as well as in human heart tissue treated with ART.

### 3.4. HOPX Regulates Cardiomyocyte Hypertrophy 

**Figure 4:**
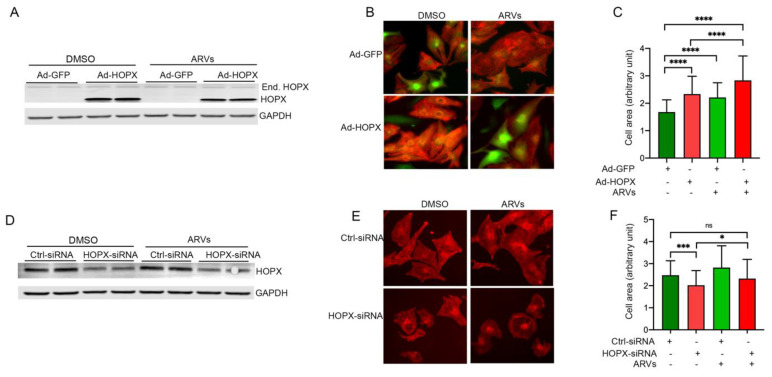
HOPX regulates ARV-induced cardiomyocytes hypertrophy. NRVCs were infected with HOPX adenovirus and treated with ARVs or DMSO for 24 h. Cardiomyocytes infected with Ad-GFP were used as control cells. (**A**) Western blot image showing HOPX protein expression in adenovirus-treated cardiomyocytes. (**B**) NRVCs were infected with Ad-HOPX or Ad-GFP and incubated for 48 h, and then treated with ARVs or DMSO for 24 h. Cells were fixed with 4% PFA and immunocytochemistry was performed with actinin antibody, and nuclei were counterstained with DAPI. Representative microscopy images showing cardiomyocytes after Ad-HOPX and ARVs treatment. (**C**) The graph shows the quantification of cardiomyocyte cell size. Cell size was determined using ImageJ software (n = 100). To knockdown the expression of HOPX in cardiomyocytes, NRVCs were treated with HOPX-siRNA or control-siRNA for 24 h, and then cells were treated with ARVs or DMSO for 24 h. Proteins were isolated for the Western blot, or cells were fixed for the microscopy. (**D**) Western blot images show a decrease in HOPX expression level in HOPX-siRNA transfected cells compared to control siRNA. (**E**) Representative microscopy images show cardiomyocytes treated with HOPX-siRNA and ARVs. Cells were stained with actinin antibody. (**F**) The graph shows the quantification of cardiomyocyte cell size. Cell size was measured using Image J software (n = 100). (*p*-value = ns, not significant; *, <0.05; ***, <0.001; ****, <0.0001).

An earlier study shows that HOPX plays an important role in cardiomyocytes proliferation and carcinogenesis through tuning the function of cardiac-specific transcription factor GATA4 [24]. Additionally, in our study, we found that ARV-treatment promotes the expression of HOPX in cardiomyocytes, as well as in the cardiac tissue of HIV patients on ART treatment (Figure 2). Therefore, we tested the role of HOPX in cardiomyocyte cell size. We used gain-in and loss-of functional approaches, using adenovirus and siRNA to determine the role of HOPX in cardiomyocyte cell size. Rat cardiomyocytes were infected with Ad-HOPX or Ad-GFP for 48 h and then treated with ARVs or DMSO for another 24 h. Expression of HOPX protein was detected by Western blotting (Figure 4A), and cell size was determined by immunocytochemistry. Adenovirus infected cardiomyocytes showed significantly increased HOPX expression level, as well as a cardiomyocyte cell size similar to ARV-treated cardiomyocytes (Figure 4B,C). Interestingly, our data show that ARVs treatment further increases the size of HOPX-overexpressing cells, which suggests that HOPX and ARV treatments have a synergistic effect on cardiomyocyte cell size (Figure 4B,C). Conversely, we found that cardiomyocytes treated with HOPX-siRNA did not increase the size of ARV-treated cardiomyocytes (Figure 4D–F). Our results suggest that HOPX plays an important role in the ARV-mediated regulation of cardiomyocytes hypertrophy.

### 3.5. HOPX Is Critically Involved in Epigenetic Modification of Histone 3 during ARV Treatment

**Figure 5:**
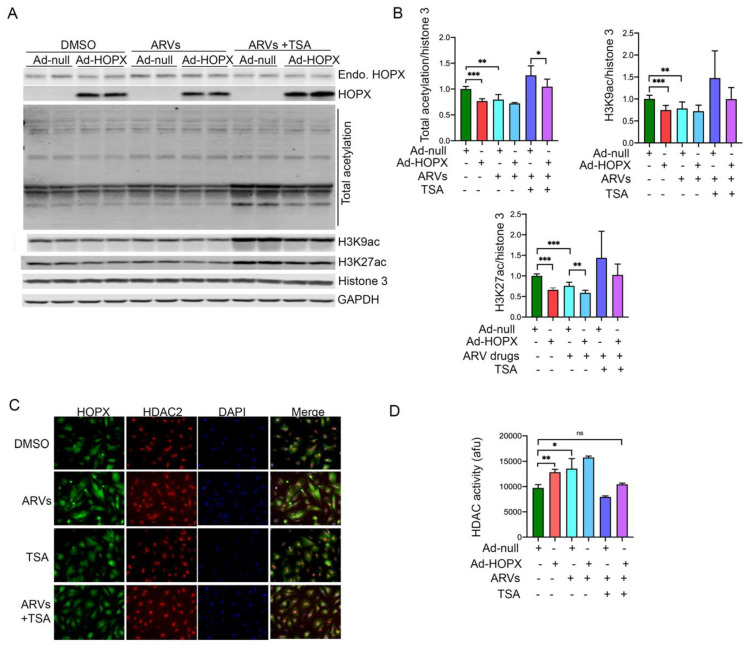
HOPX regulates ARV-mediated epigenetic modification of histone 3. ARV treatment in cardiomyocytes reduces total acetylated lysine level, as well as acetylation of histone 3 at K27 and K9. Cardiomyocytes were transduced with Ad-HOPX for 48 h and treated with ARVs, alone or in a combination with TSA (50 nM), for another 24 h. Control cells were transduced with Ad-null treated with ARVs or DMSO. (**A**) Western blot images show the expression of HOPX, total lysine acetylation, and acetylation at H3K9 and H3K27. (**B**) Graphs show quantification of Western blot images (*, *p*-value <0.05; **, <0.01; ***, <0.001). Values are presented as means ±SDs from three biological replicates and two technical replicates each time. (**C**) NRVCs were treated with ARVs or TSA for 24 h and fixed with 4% PFA. Cells were stained with HOPX and HDAC2 antibodies, and nuclei were counterstained with DAPI. Representative microscopic images of cardiomyocyte show localization of HOPX (Green), HDAC2 (Red), and nucleus (DAPI). (**D**) NRVCs were transduced with the Ad-HOPX or Ad-null and treated with the ARVs and TSA or DMSO. HDAC activity was determined using the protein lysate. Graph showing the deacetylase activity of HDAC. (*p*-value, ns = not significant; *, <0.05; **, <0.01; ***, <0.001).

It has been reported that HOPX physically interacts with the HDAC2 and regulates the acetylation level of histone 3 at the promoter region of cardiac-specific transcriptional factor GATA4, as well as inhibiting the expression of anti-hypertrophy genes [24]. Since we found that HOPX expression was increased in ARV-treated cells, we determined the total cellular lysine acetylation level of cardiomyocytes after drug treatment by Western blotting. Our Western blot data analysis shows that drug treatment significantly reduced the total cellular lysine acetylation (Figure 5A,B). In our previous study, we found that ARV-treatment regulates the epigenetic modifications of histone 3, through regulation of the acetylation at H3K9 and H3K27. In this study, we investigated the role of HOPX in ARV-induced deacetylation at H3K9 and H3K27. Interestingly, overexpression of HOPX significantly reduces the acetylation level at H3K9 and H3K27, similarly to ARV-treated cells (Figure 5A,B). Since HOPX promotes deacetylation through interaction with HDAC2, we then tested whether TSA, a pharmaceutical inhibitor of HDAC, could restore the acetylation level of cells in the presence of ARVs. Interestingly, our data show that TSA can restore total cellular lysine acetylation, as well as acetylation of H3K9 and H3K27 (Figure 5A,B). Furthermore, we performed immunocytochemistry in rat cardiomyocytes treated with ARVs, to detect the localization of HOPX and HDAC2 during ARV treatment and TSA-mediated HDAC inhibition. Representative images show that HDAC protein co-localizes with HOPX and drug treatment causes increased accumulation of HOPX in and around the nucleus (Figure 5C). Furthermore, we found that TSA treatment can reverse the effect of ARV-mediated HOPX accumulation in cardiomyocytes (Figure 5C). Since HOPX forms a functional complex with HDAC2, we performed a biochemical assay to determine the HDAC activity in cardiomyocytes overexpressing HOPX and treated with ARVs. The HDAC activity assay showed that HOPX overexpression and ARV treatment promotes the HDAC activity in cardiomyocytes, which are blunted in the presence of HDAC inhibitor TSA (Figure 5D). 

### 3.6. HOPX Is Important to Maintain an Optimum Acetylation Level in the Histone 3 

**Figure 6:**
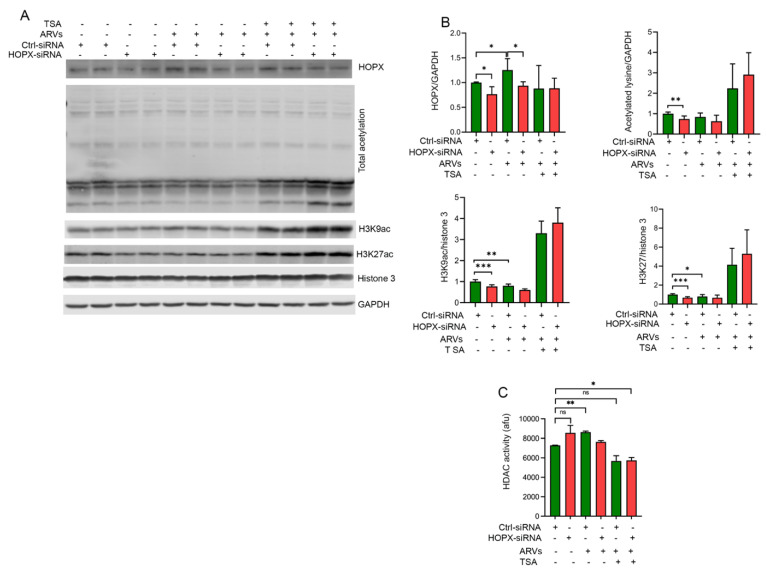
Downregulation of HOPX may cause aberrant cellular lysine acetylation. HOPX expression was knocked down using HOPX-siRNA in NRVCs for 30 h and then treated with ARVs and TSA for another 24 h. Control cells were treated with DMSO. (**A**) Western blot images showing expression of HOPX, total cellular acetylation, H3K9ac, and H3K27ac in HOPX-siRNA-treated cardiomyocytes. Furthermore, Western blots show that TSA treatment restores the total and H3K9, H3K27 acetylation in Hopx-siRNA- and ARV-treated cardiomyocytes. (**B**) Graphs show quantification of Western blot images of HOPX, total acetylated lysine, H3K9ac, and H3K27ac. Values are presented as means ±SDs from three biological replicates and two technical replicates each time. (**C**) Graph shows deacetylase activity of the HDAC in cardiomyocytes treated with HOPX-siRNA and ARVs or DMSO (*p*-value, ns = not significant; *, <0.05; **, <0.01; ***, <0.001). NRVCs were treated with the HOPX-siRNA or Ctrl-siRNA for 30 h and then treated with the ARVs and TSA for another 12 h.

We also analyzed the total cellular acetylation in cardiomyocytes treated with HOPX-siRNA. Our Western blot data show that loss in HOPX expression causes deacetylation of total cellular lysine, as well as deacetylation at H3K9 and H3K27. Decreased acetylation of siRNA-treated cells may be due to increased competitive binding of HOPX and HDAC2 or upregulation of other deacetylase enzymes. However, loss in HOPX expression level in TSA-treated cardiomyocytes can increase total cellular lysine acetylation and acetylation level at H3K9 and H3K27 (Figure 6A,B). We believe that TSA interferes with the functional enzyme complex of HDAC2 and HOPX, which results in increased cellular acetylation levels in HOPX-knockdown cells. In addition, the HDAC activity assay showed that siRNA-mediated knockdown of HOPX increases the HDAC activity in cardiomyocytes (Figure 6C). Taken together, our data show that HOPX may be a critical regulator of ARV-mediated epigenetic modification in cardiomyocytes. 

## 4. Discussion

Coronary heart disease, atherosclerosis, stroke, and myocardial infarction, in combination or alone, cause major CVDs in HIV+ patients and are responsible for the induction of comorbidity. However, the molecular and cellular mechanisms associated with ARV-induced cardiotoxicity and dysfunction remain unclear. In our previous study, we found that ARVs treatment reduces cellular viability, increases cellular oxidative stress, and promotes cellular hypertrophy. Additionally, we found that ARVs act as a transcriptional suppressor, through epigenetic modification of histone 3 in cardiomyocytes [31]. Our previous results prompted us to discover the mechanism of ARV-induced changes in cardiomyocyte viability and hypertrophy. To find out the mechanism, we treated cardiomyocytes with ARVs, and the global gene expression profile was determined using an RNA-seq experiment. Analysis of the RNA-seq data shows that several biological processes related to cell death, cell proliferation, cellular stress, and immune response were upregulated in the drug-treated cells, whereas biological processes such as cell division and energy metabolism were downregulated in the cardiomyocytes treated with ARVs. Furthermore, the analysis of differentially expressed genes shows that major transcriptional changes in the cardiomyocytes are associated with cardiotoxicity, through cardiac enlargement or hypertrophy. Several studies were performed to determine the cause of functional and structural cardiac abnormalities in HIV patients having ART [35,36,37,38,39]. Echocardiography data analysis of first-line active antiretroviral therapy patients shows that left ventricular hypertrophy and left atrial enlargement are prevalent in PLWH [35]. Additionally, it was reported that increased left ventricular mass or cardiac enlargement is associated with the use of the protease inhibitor in the cocktail of ART [38]. Our in vitro study also found that ARV-treatment promotes the gene expression associated with cardiomyocyte cell size and hypertrophy.

Since we found that cardiac enlargement was the topmost cardiotoxicity term in the RNA-seq data analysis, we validated the RNA-seq data through expression analysis of the cardiac enlargement-associated genes found to be upregulated in the ARV-treated cardiomyocytes. Our qRT-PCR data show that expression of the hypertrophy-related genes Hopx, P2rx4, and Ackr3 was significantly upregulated in ARV-treated cardiomyocytes, as well as in the cardiac tissue of HIV patients who have a history of ART. Additionally, our data also show that HOPX protein expression and distribution were significantly altered in the cardiac cells of HIV patients. Therefore, we studied the role of HOPX in ARV-mediated cardiomyocyte structure and function. 

HOPX is an atypical homeodomain-containing protein that lacks a DNA binding domain. Earlier studies show that HOPX plays a critical role in cardiac development. It acts as a co-suppressor, through interactions with several chromatin-modifying enzymes, including HDAC2. A study performed using the Hopx knockout mouse model suggests that HOPX plays a critical role in lysine deacetylation, through interaction with the deacetylase enzymes, and is important for the proper recruitment of the deacetylase enzymes with the promoter [24,40]. We evaluated the role of HOPX in cardiomyocytes, through overexpression and knockdown. Our data show that expression of HOPX significantly upregulates the cardiomyocyte hypertrophy, whereas silencing the HOPX expression causes a reduction in cell size. Furthermore, we found that ARV-treatment causes a significant increase in the cell size of HOPX-expressing cells. This finding suggests that HOPX is an important factor in the determination of cell size of differentiated cardiomyocytes during stress conditions. Similarly, a previous study suggested that HOPX forms a complex with HDAC and inhibits cardiomyocyte proliferation, through antagonizing the SRF-mediated gene transcription [41,42,43]. Since we found that ARV treatment causes a significant reduction of cellular acetylation, we evaluated the role of HOPX in ARV-mediated changes in histone 3 acetylation. Interestingly, our data show that ARV treatment, alone or ARVs with HOPX overexpression, significantly reduces the acetylation of histone protein. Furthermore, we found that HOPX causes a significant reduction of acetylation at H3K9 and H3K27. It is believed that H3K9ac and H3K27ac are the active histone markers, and the reduction of acetylation causes significant transcription repression. Our study also shows that ARVs treatment causes a significant increase in co-localization of HOPX and HDAC, which may be responsible for the ARV-mediated reduction of acetylation in H3K9 and H3K27. Furthermore, our data show that the use of the HDAC inhibitor TSA, in the presence of ARVs, preserves the total acetylation of histone and acetylation of H3K9 and H3K27.

In summary, we attempted to understand the molecular mechanism of cardiac hypertrophy during ARV treatment. We found that several hypertrophy-related gene expressions were significantly upregulated due to ARV treatment. Additionally, we identified that the expression of a cardiac-specific transcription factor HOPX was upregulated due to drug-mediated cellular stress. HOPX acts as a transcriptional suppressor, which regulates the expression of various anti-hypertrophy genes, through interaction with the deacetylase enzyme HDAC [40]. HOPX interaction with HDAC may also be responsible for ARV-induced epigenetic modification and suppression of gene transcription. 

## 5. Conclusions

HOPX may play a critical role in ARV-induced epigenetic modification, as well as cardiac hypertrophy, through interacting and modulating the function of HDAC. Thus, TSA could be used as a potential therapeutic agent to treat cardiac hypertrophy in PLWH.

## Data Availability

The data presented in this study are available upon request from the corresponding author.

## Supplementary Materials

The following are available online at https://www.mdpi.com/article/10.3390/cells10123458/s1, Supplementary Table S1: Identification of differentially expressed genes in ARVs treated NRVCs; Supplementary Table S2: Identification of upregulated biological processes associated with ARVs treatment in NRVCs; Supplementary Table S3: Identification of downregulated biological processes associated with ARVs treatment in NRVCs; Supplementary Table S4: Differentially expressed genes in ARVs treated cardiomyocytes are associated with cardiac enlargement and hypertrophy; Supplementary Table S5: List of primers used for qRT-PCR analysis; Supplementary Table S6: Patient information.
