# Supplementary material for: HOPX Plays a Critical Role in Antiretroviral Drugs Induced Epigenetic Modification and Cardiac Hypertrophy
Source: Cells. 2021 Dec 8;10(12):3458. doi: 10.3390/cells10123458 (PMC8700328; doi:10.3390/cells10123458)
Supplement: Supplementary file 1 [file cells-10-03458-s001.zip › Supplementary Table S5.pdf]

Supplementary Table S5 : Genes specific primers used in qRT-PCR

Genes	Primer Sequence (5'-3')
P2rx4 Rat F	GTA TGT GGA AGA CTA CGA GCA G
P2rx4 Rat R	CTC TCC CCA TCT TTC TGC TTC
Hopx Rat F	TCC GTT TCA GAG CAT TCC AG
Hopx Rat R	ACC CGT GTT CTC ATC CAA C
Ackr3 Rat F	AGC ACT CAA AGC CAG GAA G
Ackr3 Rat R	GCC ATG TGA TGT CCG AGT AG
P2RX4 Human F	TCA CCA TGA ACC AGA CAAC AG
P2RX4 Human R	ACA GAC CCG TTG AAA GCT AC
ACKR3 Human F	GGC CTT CAT CTT CAA GTA CTC G
ACKR3 Human R	CAA GTA AAC CCG TCC CAG AG
HOPX Human F	ATG ATA GTC TGC CGT GCT TG
HOPX Human R	CTG CCA TCT CCT TAG TCT GTG
