# Supplementary material for: HOPX Plays a Critical Role in Antiretroviral Drugs Induced Epigenetic Modification and Cardiac Hypertrophy
Source: Cells. 2021 Dec 8;10(12):3458. doi: 10.3390/cells10123458 (PMC8700328; doi:10.3390/cells10123458)
Supplement: Supplementary file 1 [file cells-10-03458-s001.zip › Supplementary Table S6.pdf]

Supplementary Table S6: Patient's information

Patients No	HIV status	Age	Race	Risk	ART	Viral load copies/ml	CD4
1	-	50-60	H	NA	-	NA	NA
2	-	50-60	B	NA	-	NA	NA
3	-	50-60	B	NA	-	NA	NA
4	-	50-60	H	NA	-	NA	NA
5	-	40-50	W	NA	-	NA	NA
6	-	60-70	H	NA	-	NA	NA
7	-	60-70	B	NA	-	NA	NA
8	-	30-40	H	NA	-	NA	NA
9	+	60-70	B	IVDU	+	30	137
10	+	60-70	H	SEX	+	undetected (<20)	1808
11	+	70-80	W	NA	+	undetected (<20)	472
12	+	60-70	B	IVDU	+	380	158
13	+	50-60	B	SEX	+	undetected (<50)	42
14	+	60-70	B	SEX	+	undetected (<20)	55
15	+	NA	NA	NA	+	NA	NA
16	+	NA	NA	NA	+	NA	NA
17	+	NA	NA	NA	+	NA	NA
18	+	NA	NA	NA	+	NA	NA
19	+	NA	NA	NA	+	NA	NA
